# What is important to the GP in recognizing acute appendicitis in children: a delphi study

**DOI:** 10.1186/s12875-023-02167-6

**Published:** 2023-10-23

**Authors:** Guus C.G.H. Blok, Marjolein Y. Berger, Arjan B. Ahmeti, Gea A. Holtman

**Affiliations:** Department of General Practice and Elderly Care Medicine, University Medical Center Groningen, University of Groningen, PO Box 196, Groningen, 9700 AD The Netherlands

**Keywords:** Primary Health Care, Appendicitis, Child, Abdominal Pain, Delphi Technique, Medical Records

## Abstract

**Background:**

For diagnostic research on appendicitis in registration data, insight is needed in the way GPs generate medical records. We aimed to reach a consensus on the features that GPs consider important in the consultation and medical records when evaluating a child with suspected appendicitis.

**Methods:**

We performed a three-round Delphi study among Dutch GPs selected by purposive sampling. An initial feature list was created based on a literature search and features in the relevant Dutch guideline. Finally, using a vignette describing a child who needed later reassessment, we asked participants to complete an online questionnaire about which consultation features should be addressed and recorded.

**Results:**

A literature review and Dutch guideline yielded 95 consultation features. All three rounds were completed by 22 GPs, with the final consensus list containing 26 symptoms, 29 physical assessments and signs, 2 additional tests, and 8 further actions (including safety-netting, i.e., informing the patient about when to contact the GP again). Of these, participants reached consensus that 37 should be actively addressed and that 20 need to be recorded if findings are negative.

**Conclusions:**

GPs agreed that negative findings do not need to be recorded for most features and that records should include the prognostic and safety-netting advice given. The results have implications in three main domains: for research, that negative findings are likely to be missing; for medicolegal purposes, that documentation cannot be expected to be complete; and for clinical practice, that safety-netting advice should be given and documented.

**Supplementary Information:**

The online version contains supplementary material available at 10.1186/s12875-023-02167-6.

## Introduction

Recognizing appendicitis in children can be challenging for general practitioners (GPs) [1]. Diagnostic tools available for secondary care could assist GPs, but require validation for use in primary care [2]. While prospective studies are costly and time-consuming due to the low prevalence of appendicitis, registration data studies may be a feasible alternative for validation [3, 4]. However, registration database research has methodological drawbacks due to the fact that medical records were not primarily intended for research, but to support memory and communication with colleagues [5]. The same constraint applies to other purposes of medical records like decision support and medicolegal issues [6, 7]. Notably, registration data are likely to suffer from missing data since clinicians use pattern recognition for their assessment and stop collecting information when new information would not change their decision anymore [5, 8]. This may lead to biased results whenever missingness of data cannot be explained by other variables in the database [2, 5, 9]. Knowing which information GPs consider important to be recorded could improve data interpretation. Therefore, we conducted a Delphi study among GPs to reach consensus on which features are important to address and record when evaluating a child with suspected appendicitis.

## Methods

### Study design

We invited GPs to engage in a modified Delphi procedure with a predefined feature set designed to seek consensus on the information that should be addressed and recorded for a child with suspected appendicitis. Participants received a vignette that described a typical child with suspected appendicitis who needed later reassessment by a second clinician in a primary care setting (Box 1).

#### Development of feature list

We identified the initial list of putative consultation features from two sources. First, we conducted a systematic review of appendicitis in primary care based on two literature searches in PubMed, conducted from inception to 28th August 2019, focusing on clinical results (i.e., symptoms, signs, and tests), medical reporting, and safety-netting advice (Appendix 1). Second, we used the Dutch guideline for GPs to add relevant features missing from the reviews [10]. We then discussed the applicability of each feature and categorized them as a symptom, sign, additional test, diagnosis, or action (including safety-netting advice, i.e., instructing to patients when to seek further medical attention [11]). Thereafter, we considered the need to address a given feature and record its presence or absence.

### Expert panel

The expert panel comprised GPs selected by purposive sampling from GPs in the northern Netherlands to ensure a diverse group with respect to gender, age, clinical experience (years in practice), and research experience [12]. We aimed to include 12–20 GPs based on a priori consensus and invited participants with an e-mail that included information about the study objectives [13].

### Data collection and analysis: the delphi process

All analysis was done using IBM SPSS for Windows, version 25.0 (IBM Corp., Armonk, NY, USA). We set the maximum number of Delphi rounds to three to improve compliance [13]. The expert panel members received an online questionnaire (Qualtrics, Provo, UT, USA) at the start of each round.

In Round 1, we recorded the characteristics of panel members and asked three questions: “Is the feature important in the consultation?”, “Is it important to record the feature’s *presence*?”, and “Is it important to record the feature’s *absence*?”. When they could not comment on the presence or absence of a feature (e.g., pain location, safety-netting advice), we asked two additional questions—“Is the feature important in the consultation?” and “Is it important to record the findings or actions?”—with importance rated on 5-point Likert scales (i.e., not important at all, not important, neutral, important, very important). In the Netherlands, GP records are electronic and accessible to patients and to other GPs who provide care to the patient, also outside regular hours. We asked panel members to comment on the questionnaire and to suggest new features for the next round. The results were then analyzed for consensus, discussed by the authors, and used to adjust the questionnaire for the next round. Finally, we generated a revised concept consensus list with features rated important or very important by ≥ 70% of participants and removed features rated not important at all, not important, or neutral by ≥ 70%.

In Rounds 2 and 3, we encouraged participants to reassess their initial judgment about the importance of the required consultation features by presenting them with the percentages given for each requirement in the previous round and asking them if they considered it important (yes/no). We also asked them to rate, by five-point Likert scale, the importance of any requirement that had either been added or that had not reached consensus in the prior round. Requirements that were newly rated as important or very important by ≥ 70% of participants were placed on the concept consensus list after each round. After Round 2, we included requirements from the concept consensus list in the final consensus list if ≥ 70% of participants agreed on their importance. After each round, we removed requirements rated as important or very important twice by < 70% of participants, as well as those rated not important at all, not important, or neutral by ≥ 70% of participants.

The final consensus list included all features and associated requirements ranked by the degree of consensus. Specifically, we ranked the symptoms, signs, and additional tests on a feature’s importance and on the importance of recording its presence or absence, and we ranked diagnoses and GP actions (including safety-netting advice) on the need to act and record that action. Additionally, we report the qualitative feedback of participants for each feature, unless it merely repeated the answer in the questionnaire.

## Results

### Expert group

Of the 33 expert panel members, 22 (67%) completed the first, second, and third rounds. Participants comprised 9 (41%) men and had a median age of 43 years (35–56 years, Q1–Q3) and a median of 14 years’ experience (4–23 years, Q1–Q3) in general practice (Table 1).

**Table 1:** Characteristics of the expert panel participants

Characteristic	Participants (n = 22)
*Gender*	
Male, *n (%)*	9 (41%)
Female, *n (%)*	13 (59%)
*Age (years), median (Q1–Q3)*	44 (35–56)
30–40, *n (%)*	9 (41%)
41–50, *n (%)*	6 (27%)
51–60, *n (%)*	4 (18%)
60–70, *n (%)*	3 (14%)
*Years in practice, median (Q1–Q3)*	14 (4–23)
1–10, *n (%)*	10 (45%)
11–20, *n (%)*	6 (27%)
21–30, *n (%)*	3 (14%)
31–40, *n (%)*	3 (14%)
*Days in workweek, median (IQR)*	3 (1.5)
2, *n (%)*	2 (9%)
2.5, *n (%)*	3 (14%)
3, *n (%)*	6 (27%)
3.5, *n (%)*	3 (14%)
4, *n (%)*	8 (36%)
*Experience in research*	
None, *n (%)*	5 (23%)
Little, *n (%)*	12 (54%)
Much, *n (%)*	5 (23%)

### Feature list

As summarized in Appendix 2, we identified 332 papers that matched the search criteria and selected 18 after content review [10, 14–29]. Appendix 3 summarizes the aims and features of the included papers. Together with the features detailed in the Dutch guideline, the initial list included 95 features and 217 associated requirements (Appendix 4).

### Consensus list

Appendix 5 shows the percentages of experts considering requirements important or not in each Delphi round. Participants added 8 features and 11 associated requirements in round 2, resulting in a list of 103 features and 228 associated requirements. Additionally, we removed 37 features and 129 associated requirements that lacked consensus. The final consensus list therefore comprised 66 features with 99 associated requirements: 26 symptoms (48 requirements), 29 physical assessments and signs (36 requirements), 2 additional tests (3 requirements), 1 possible diagnosis (2 requirements), and 8 GP actions, including safety-netting advice (10 associated requirements). Table 2 summarizes the included symptoms and associated requirements, while table 3 summarizes the physical assessments and signs.

**Table 2:** Final ranked consensus on symptoms

Feature: Symptoms	Requirement			
	Ask	Record present	Record absent	Record finding
Location of abdominal pain	100			**100**
Abdominal pain	100	100	**96**	
Duration of pain	96			
Fever	96	100	**96**	
Abnormal menstrual cycle	96	86		
Pregnancy	91	100	**77**	
Vomiting	91	100		
Constipation	91	100		
Diarrhea	91	100		
Transportation pain	91	100	**73**	
Intensity of pain	86			
Prior abdominal operation	86	91		
Type of pain	82			**86**
Use of analgesics	82			
Opioids		91		
NSAIDs		91		
Paracetamol		86		
Whether pain is acute or chronic	77			**86**
Nausea	77	86		
Migration of pain	77			
Differential diagnosis UTI	77			
Dysuria	76	91		
Sexual risk behavior	73	77		
Blunt abdominal trauma		100		
Rectal bleeding		91		
Vaginal bleeding		91		
Hematuria		91		
Chronic disease		82		
Frequent micturition		73		

The 7 requirements in **bold** are considered important even when all items are negative. Figures represent consensus percentages (≥ 70%). Abbreviations: NSAID, non-steroidal anti-inflammatory drug; UTI, urinary tract infection

**Table 3:** Final ranked consensus on physical assessments, signs, and additional tests

Feature	Requirement			
	Examine	Record present	Record absent	Record finding
**Sign**				
General appearance	100			
Drowsiness		100		
Ill appearance		100	**96**	
Jaundice		96		
Palpation	100			
Location of tenderness				**100**
Guarding		100	**77**	
Peritoneal irritation		100	**77**	
Abdominal mass		100		
Tenderness		96	**82**	
Rebound tenderness		96	**73**	
Fecal mass		96		
Measurement of temperature	96			**100**
Have location of pain pointed out	96			
Difficulty walking	82	73		
Inability to walk	82	86		
Auscultation	82			
Normal bowel sounds			**100**	
Inspection	77			
Presence of scars		77		
Abdominal distension		82		
Purpura		96		
Percussion	77			
Tenderness on percussion		82		
Jaundice		96		
Scrotal swelling/tenderness		96		
Psoas sign		86		
Purpura		86		
Summarize as “abdomen completely soft without tenderness”				**86**
**Additional test**				
Urinalysis	94			
CRP-POCT	77			**91**

Abbreviations: CRP, C-reactive protein; POCT, Point-of-care testing

The 10 requirements in **bold** are considered important when all items are negative. Figures represent consensus percentages (≥ 70%)

#### Symptoms, signs, and additional tests

GPs reached full consensus (100%) on the need to ask about abdominal pain and the location of that pain, as well as the need to record a history of vomiting, diarrhea, constipation, fever, pregnancy, transportation pain, and blunt abdominal trauma. They also reached full consensus on the need for general examination, abdominal palpation, and documenting the presence of drowsiness, “ill” appearance, guarding, peritoneal irritation, and abdominal mass. Consensus existed on summarizing a normal abdominal examination as “abdomen completely soft without tenderness” (86%) and on the importance of point-of-care testing (POCT) for urinalysis (94%) and C-reactive protein (CRP) (77%). This included the need to record the CRP level (91%).

#### Possible diagnoses

Consensus existed that assessment should include an ICPC (International Classification of Primary Care) symptom code rather than a diagnostic code for appendicitis (86%) and that GPs should record a differential diagnosis (77%) (Table 4).

**Table 4:** Final ranked consensus on diagnosis and actions

Feature	Requirement	
**Diagnosis**	Action*	Record
ICPC symptom code		86
differential diagnosis		77
**Actions**		
Find help when needed	91	77
Alarm symptoms	91	73
How to find help	91	
Uncertainty of diagnosis	91	
How follow-up will take place	82	
Peer consultation		86
Options for follow-up		81
Expected course		77

Abbreviations: ICPC, International Classification of Primary Care

Figures represent consensus percentages

*Actions by the GP, such as “discuss” or “communicate.”

#### GP actions

Participants agreed that GPs should discuss potential alarm symptoms during the consultation (91%) and instruct patients to seek help when needed (91%), with consensus on the need to record both (73% and 77%, respectively) (Table 4). There was also consensus that GPs should explain uncertainty about the expected course (91%) and how to find help (91%). Furthermore, participants agreed that GPs should record any peer consultation (86%), planned follow-up (82%), discussion about follow-up options (81%), and advice for the next physician (77%).

#### Minimum reporting requirements

Of the 66 features on the final consensus list, 52 required recording and 37 required actions (ask/examine/explain) when addressed during the consultation. Finally, we identified a minimum of 20 features that needed to be recorded if findings were negative (7 Symptoms, 8 physical assessments and signs, 1 test, 2 diagnoses, 2 actions; Tables 2, 3 and 4, in bold). Of note, the qualitative feedback revealed that GPs do not routinely address all features, instead focusing on those that attract attention or have relevance to the differential diagnosis (Appendix 6).

## Discussion

### Strengths and limitations

This study had a high response rate, with almost 70% of the invited GPs agreeing to participate in, and then completing, the three Delphi rounds. This suggests that few participants were likely to have a special interest in the subject [13]. As shown in Table 1, our sample included a diverse cross-section by experience, sex, and age. However, cultural, geographical, or educational backgrounds were not used as criteria for the purposive sampling, which could limit the diversity of perspectives represented in the study. The inclusion of 22 participants was in accordance with recommendations found in the literature [13]. Furthermore, our research questions relied on the subjective opinions of experts, resulting in a long consensus list that may not reflect routine practice [13]. The modified Delhi procedure may have caused this lengthy output due to the inclusion of a comprehensive list in the first round, which we designed to avoid biasing the responses of participants. By contrast, a classic procedure without an initial list (i.e., adding features using open questions) might have yielded a different final consensus list. The consensus list can serve as starting point for further research. However, a more concise list would have benefitted clinicians, which would have required extending the Delphi procedure with one or more rounds. The requirement for reassessment in our vignette also implies uncertainty about the diagnosis, which may have resulted in the respondents hesitating to label a clinical feature as unimportant [5]. Performing a study with a different level of uncertainty could prove interesting and may yield a different list. Finally, we did not offer background information on the diagnostic value of the clinical features of appendicitis. Although this might have yielded a more substantiated consensus list, we did not want to influence the participants’ opinions in this way [30].

GPs in this study fully agreed on the importance of recording the location of any pain or tenderness, whereas a cohort study of registration data for children with acute abdominal pain revealed that only 29% recorded findings on right lower quadrant pain and that only 55% recorded tenderness [1]. These findings suggest that a discrepancy exists between what GPs consider important and what is actually implemented in practice, meaning that missing values in registration data cannot be fully explained by our results. This finding also suggests that GP select features to be assessed and recorded from a vast range of features in their cognition which is consistent with the use of illness scripts for pattern recognition.

### Comparison with existing literature

Other researchers have used the Delphi method to reach consensus on medical reporting in primary and secondary care [31, 32]. A Delphi study on the content of medical reporting by endoscopists could not specify the diagnostic information required for such a report, [33] consistent with our findings and the intuition-based or informal diagnostic approaches used by GPs [34]. Therefore, statistical analysis of the predictive values of clinical features (e.g., in the shape of a prediction model), will benefit from standardized reporting based on a shorter and more practical list of clinical features for reporting.

Notably, participants agreed on the need to request CRP-POCT (78%), commenting that CRP is widely used when managing suspected appendicitis in children. Although the Dutch GP guideline does not support using CRP, due to a lack of evidence that it adds value above symptoms and signs of appendicitis among children in primary care, [10] a recent study of registration data has shown that a CRP test result does add value in this setting [4]. However, in the Netherlands CRP-POCT is available to GPs, whereas White Blood Counts (WBC) are available but not as point-of-care-testing, which could have had an impact the results. Consensus also existed on the need to record safety-netting advice, including the existence of prognostic uncertainty, the alarm symptoms that warrant further assessment, how to find help, and advice about the prognosis [18]. Given that GPs do not universally record safety-netting advice in their current practice, [6] this represents a prime target for improvement.

### Implications for research and/or practice

Our findings have implications for research, medicolegal reporting, decision-making, and access to medical records by out-of-hours care providers and by patients.

For research, the greater likelihood of recording positive than negative findings is incompatible with the assumption of data missing completely at random [2, 5]. Further studies could assess what imputation method (e.g., multiple imputation and zero imputation) produces least bias in datasets where negative findings are more likely to be missing than positive findings [1].

When accounting for care given in medical negligence settings, our results contradict the assumption “if it is not documented, it did not happen,” because the absence of features does not necessarily indicate that care has not been given. Given the consensus on the need to document safety-netting advice by the expert panel in this study, our results could establish a norm for this standard of care [35].

Evidently, the length of our consensus list makes it unsuitable for routine use in support of clinical decision-making in practical guidelines that cover all situations where the differential diagnosis includes appendicitis. Therefore, we suggest clinical guidelines should state the diagnostic value of the most important clinical features relevant to the differential diagnosis, rather than specifying unrealistically long lists of clinical features that should be assessed [36]. A clinical prediction rule for use in primary care would help to make the decision-making process more uniform and effective [37]. Several validated rules already exist for appendicitis in secondary care (e.g. Alvarado, AIR score), but none have been developed specifically for use in primary care [38]. Such a clinical prediction rule could be helpful when a second clinician reassesses the patient by helping to ensure that the most important features are compared. Finally, consensus on the importance of recording safety-netting and other advice may indicate that patients could benefit from signposting to this advice in their electronic health records at the end of a consultation [6].

## Conclusion

We conducted a Delphi study to identify consensus among GPs on the recommended consultation items and medical records of children who present with suspected appendicitis. GPs agreed that negative findings do not need to be recorded for most features and that records should include the prognostic and safety-netting advice given. The results have implications in three main domains: for research, that negative findings are likely to be missing; for medicolegal purposes, that documentation cannot be expected to be complete; and for clinical practice, that safety-netting advice should be given and documented.

### Box 1

GPs were asked to answer the following question for each consultation feature:

What information should be obtained and recorded for a child with suspected appendicitis when later re-assessment by a second GP may be necessary? The suspicion of appendicitis is too low to warrant immediate referral to secondary care.

### Electronic supplementary material

Below is the link to the electronic supplementary material.

Supplementary Material 1

Supplementary Material 2

Supplementary Material 3

Supplementary Material 4

Supplementary Material 5

Supplementary Material 6

## Data Availability

Datafiles are available on request from the corresponding author (g.a.holtman@umcg.nl).

## Abbreviations

CPR: Clinical prediction rule
CRP: C-reactive protein
ICPC: International Classification of Primary Care
POCT: Point-of-care test
UTI: Urinary tract infection
