# Supplementary material for: What is important to the GP in recognizing acute appendicitis in children: a delphi study
Source: BMC Prim Care. 2023 Oct 23;24:217. doi: 10.1186/s12875-023-02167-6 (PMC10591392; doi:10.1186/s12875-023-02167-6)
Supplement: Supplementary file 1 — Supplementary Material 1 [file 12875_2023_2167_MOESM1_ESM.docx]

**Appendix 1.** Literature search strategy

**Symptoms and signs:** *("Appendicitis"[Mesh] OR appendicitis[tiab])* ***AND*** *("Child"[MeSH] OR "Adolescent"[Mesh] OR "Infant"[Mesh] OR child*[tiab] OR adolescen*[tiab] OR infan*[tiab] OR pediatr*[tiab] OR paediatr*[tiab] OR school*[tiab] OR boys[tiab] OR girls[tiab] OR teen*[tiab] OR kids[tiab] OR youth[tiab] OR neonat*[tiab])* ***AND*** *("Diagnosis"[Mesh] OR diagnos*[tiab] OR clinical decision rule*[tiab] OR clinical prediction rule*[tiab] OR CDR*[tiab] OR CPR*[tiab] OR score*[tiab] OR presentation*[tiab] OR examination*[tiab] OR suspicion*[tiab] OR predict*[tiab] OR "Risk" [MeSH] OR Risk*[tiab] OR Risk factor*[tiab] OR history*[tiab] OR “Signs and Symptoms”[MeSH ]OR symptom*[tiab] OR signs [tiab] OR standard*[tiab] OR criteria[tiab] OR guideline*[tiab])*

**Safety netting:** *(“Safety Netting”[tiab] OR Safety-netting [tiab])* ***AND*** *("General Practitioners"[Mesh] OR general practitioner*[all fields] OR “Physicians, Primary Care” [MeSH] OR Physicians, Primary Care [all fields] OR general practice [all fields] OR General Practice Physician*[tiab] OR “Primary Health Care” [MeSH] OR Primary Care*[all fields] OR primary health care [all fields])*

**Contents of report:** *("General Practitioners"[Mesh] OR general practitioner*[tiab] OR “Physicians, Primary Care”[MeSH] OR Physicians, Primary Care [tiab] OR general practice [tiab] OR General Practice Physician*[tiab] OR “Primary Health Care”[MeSH] OR Primary Care*[tiab] OR primary health care[tiab])* ***AND*** *("Medical Records"[Mesh] OR medical record*[tiab] OR medical report*[tiab] OR patient report*[tiab] OR patient record*[tiab] OR medical transcription*[tiab] OR electronic medical records[tiab] OR EMR[tiab])* ***AND*** *(data quality[tiab] OR information quality[tiab])*
