# Supplementary figures and images for: What is important to the GP in recognizing acute appendicitis in children: a delphi study
Source: BMC Prim Care. 2023 Oct 23;24:217. doi: 10.1186/s12875-023-02167-6 (PMC10591392; doi:10.1186/s12875-023-02167-6)

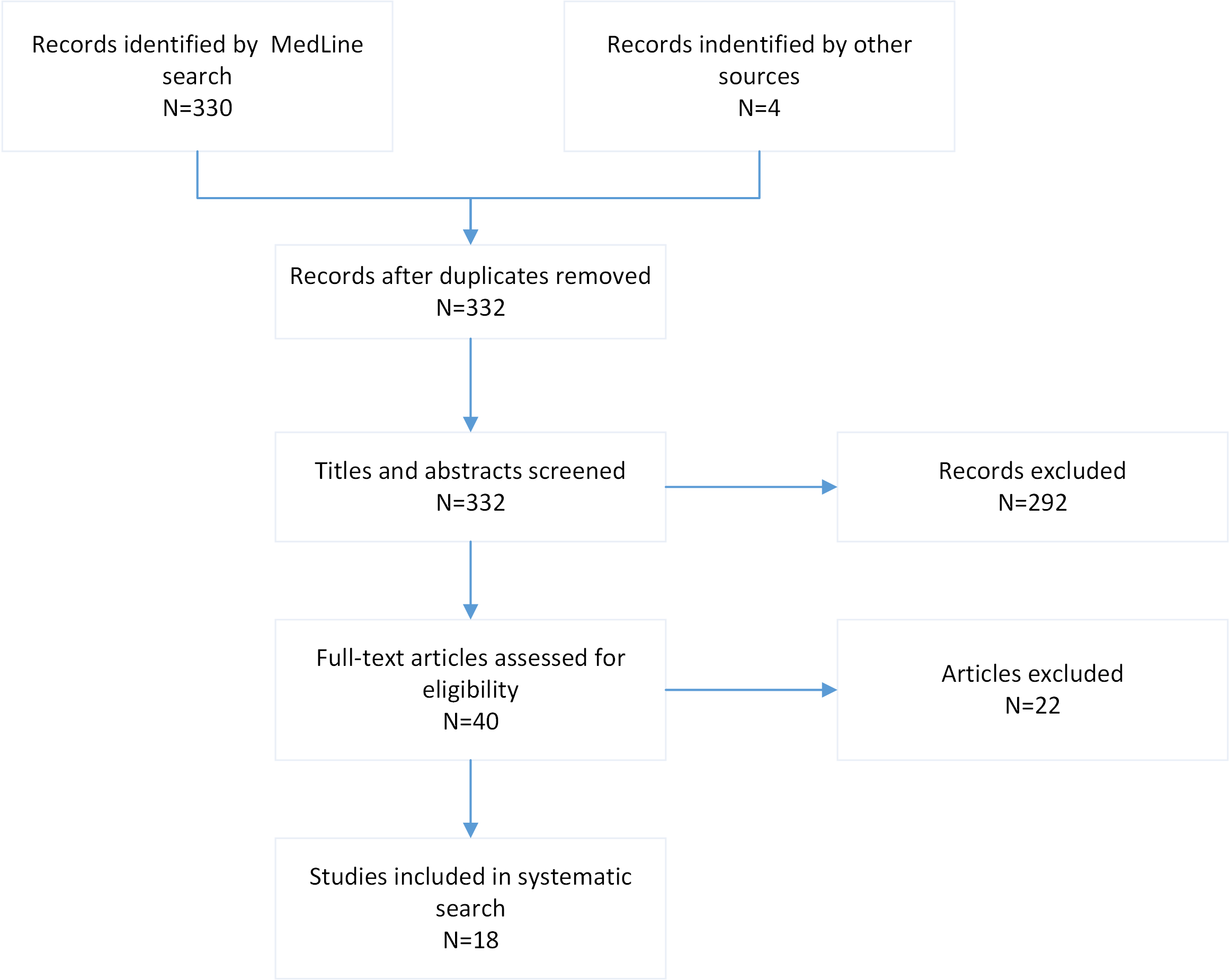

Supplement: Supplementary file 2 — Supplementary Material 2 [file 12875_2023_2167_MOESM2_ESM.png]
