# Supplementary material for: What is important to the GP in recognizing acute appendicitis in children: a delphi study
Source: BMC Prim Care. 2023 Oct 23;24:217. doi: 10.1186/s12875-023-02167-6 (PMC10591392; doi:10.1186/s12875-023-02167-6)
Supplement: Supplementary file 3 — Supplementary Material 3 [file 12875_2023_2167_MOESM3_ESM.docx]

**Appendix 3**. Results of literature search

| First author | Year | Country | Type of study | Purpose of study |
| --- | --- | --- | --- | --- |
| Benabbas, R (14) | 2017 | UK | Systematic review and meta-analysis | To evaluate which element (s) of history, physical examination, laboratory tests, PAS, or ED‐POCUS are most useful in the diagnosis of AA in ED pediatric patients. |
| Ebell, H (15) | 2014 | USA | Systematic review and meta-analysis | The objective of this study is to systematically review the accuracy of the Alvarado score and Pediatric Appendicitis Score and to identify optimal cut-offs for low- and high-risk populations. |
| Giordiano, S (16) | 2013 | Finland | Meta-analysis | To determine the value of serum bilirubin measurement in diagnosing acute appendiceal perforation. |
| Hajibandh, S (17) | 2019 | USA | Systematic review and meta-analysis | To investigate whether Neutrophil-to-lymphocyte ratio can predict acute appendicitis and whether it can distinguish between uncomplicated and complicated appendicitis. |
| Kulik, DM (18) | 2012 | USA | Systematic review | To systematically identify clinical prediction rules for children with suspected appendicitis and compare their methodological quality and performance. |
| Ohle, R (19) | 2011 | USA | Systematic review and meta-analysis | To perform a systematic review and meta-analysis of validation studies that assess the Alvarado score to determine its performance. |
| Otan, E (20) | 2013 | Turkey | Systematic review | To determine the clinical and demographic features of acute amoebic appendicitis by reviewing the reported cases. |
| Rentea, RN (21) | 2017 | Germany | Systematic review | To review the current “state of the art” in the evaluation and management of pediatric appendicitis. |
| Sharwood (22) | 2009 | Australia | Systematic review | The question of whether opioid analgesia should be given in patients with undifferentiated acute abdominal pain has been characterized by concerns about its efficacy and that signs used to determine accurate diagnosis may be masked by the drug. The objective of this review is to critically analyse pertinent pediatric randomized controlled studies considering this requirement. |
| Toumi, Z (23) | 2010 | UK | Systematic review, case reports | We present a systematic review of appendicitis following blunt abdominal trauma. The aim of this review was to collate and report the clinical presentations and experience of such cases. |
| Heurn van, LW (39) | 2014 | Netherlands, Sweden | Systematic review | The present review provides an overview of general symptoms of abdominal emergencies in children, and discusses the most common and the most devastating disorders that cause acute abdomen. |
| Dahabre, IJ (24) | 2015 | USA | Systematic review | To identify primary research studies meeting our criteria for cohort studies that reported information on test accuracy for the diagnosis of acute appendicitis or harms, and for comparative studies (randomized or nonrandomized) that reported information on patient-relevant outcomes and resource use. |
| Eizenga, WH (10) | 2012 | Netherlands | Guideline | N/a |
| Alam, R (25) | 2017 | UK | Systematic review | To synthesize the strategies, skills or traits associated with or used by clinicians working in general medical practice to manage diagnostic uncertainty and identify any existing training programs that aim to support clinicians to manage diagnostic uncertainty. |
| Almond, S (26) | 2009 | UK | Modified Delphi | In the absence of published evidence, we sought clinical consensus about safety-netting in children with acute illness. |
| Vos – Kerkhof, de E (40) | 2016 | Netherlands | Systematic review | To systematically identify evaluated safety-netting strategies after ED discharge and to describe determinants of pediatric ED revisits. |
| Jones, CH (28) | 2013 | UK | Qualitative study | We aimed to understand what safety-netting advice first contact clinicians give parents of acutely sick young children, how, when, and why. |
| Jones, D (29) | 2019 | UK | Review | To collate and summarize the evidence on safety-netting for all patients. |

Abbreviations: AA, acute appendicitis; ED, emergence department; PAS, pediatric appendicitis score; POCUS, Point-of-care ultrasound; UK, United Kingdom; USA, United States of America.
