# Supplementary material for: What is important to the GP in recognizing acute appendicitis in children: a delphi study
Source: BMC Prim Care. 2023 Oct 23;24:217. doi: 10.1186/s12875-023-02167-6 (PMC10591392; doi:10.1186/s12875-023-02167-6)
Supplement: Supplementary file 4 — Supplementary Material 4 [file 12875_2023_2167_MOESM4_ESM.docx]

**Appendix 4.** Complete feature list at the start of Delphi study

|  | Alam, R | Almond, S | Benabbas, I | Dahabre, IJ | Ebell, H | Eizenga, WH | Giordiano, S | Hajibandh, S | Heurn van, LW | Jones, CH | Jones, D | Kulik, DM | Ohle, R | Otan, E | Rentea, RN | Sharwood | Toumi, Z | Vos – Kerkhof, de E |
| --- | --- | --- | --- | --- | --- | --- | --- | --- | --- | --- | --- | --- | --- | --- | --- | --- | --- | --- |
| Abdominal pain |  |  | x |  |  | x |  |  |  |  |  |  |  |  |  |  |  |  |
| Analgesics use |  |  |  |  |  |  |  |  |  |  |  |  |  |  |  | x |  |  |
| Anorexia |  |  | x | x | x | x |  |  |  |  |  | x |  |  | x |  | x |  |
| Blunt abdominal trauma |  |  |  | x |  | x |  |  |  |  |  |  |  |  |  |  | x |  |
| Chronic disease |  |  |  | x |  | x |  |  | x |  |  |  |  | x |  |  |  |  |
| Constipation |  |  |  | x |  | x |  |  | x |  |  |  |  |  |  |  |  |  |
| Consult records of previous consultation |  |  |  |  |  |  |  |  |  |  | x |  |  |  |  |  |  |  |
| Coughing pain |  |  | x | x | x |  |  |  |  |  |  | x |  |  | x |  |  |  |
| Diarrhea |  |  |  | x |  | x |  |  | x |  |  |  |  |  |  |  |  |  |
| Duration of pain |  |  | x | x |  | x | x |  | x |  |  | x |  | x | x | x |  |  |
| Dysmenorrhea |  |  |  | x |  | x |  |  |  |  |  |  |  |  |  |  |  |  |
| Dysuria |  |  | x | x |  | x |  |  | x |  |  |  |  |  |  |  |  |  |
| Fecal incontinence |  |  |  |  |  | x |  |  |  |  |  |  |  |  |  |  |  |  |
| Fever |  |  | x | x | x | x | x |  |  |  |  |  | x | x | x |  |  |  |
| Fluid intake |  |  |  |  |  | x |  |  |  |  |  |  |  |  |  |  |  |  |
| Frequency of bowel movements |  |  |  |  |  | x |  |  |  |  |  |  |  |  |  |  |  |  |
| Frequent micturition |  |  |  |  |  | x |  |  |  |  |  |  |  |  |  |  |  |  |
| Hematuria |  |  |  | x |  | x |  |  |  |  |  |  |  |  |  |  |  |  |
| Illness in family† |  |  |  |  |  |  |  |  |  |  |  |  |  |  |  |  |  |  |
| Irritable bowel syndrome |  |  |  | x |  |  |  |  |  |  |  |  |  |  |  |  |  |  |
| Intensity of pain |  |  |  | x |  | x |  |  |  |  |  | x |  |  |  | x |  |  |
| Joint pain |  |  |  |  |  | x |  |  |  |  |  |  |  |  |  |  |  |  |
| Last use of analgesics |  |  |  | x |  |  |  |  |  |  |  |  |  |  |  | x |  |  |
| Location of pain |  |  |  | x |  | x |  |  | x |  |  |  |  |  |  |  |  |  |
| Low back pain |  |  |  |  |  | x |  |  |  |  |  |  |  |  |  |  |  |  |
| Menstrual cycle abnormal |  |  |  |  |  | x |  |  |  |  |  |  |  |  |  |  |  |  |
| Migration of pain |  |  | x | x | x | x | x |  |  |  |  | x | x |  | x |  |  |  |
| Nature of pain† |  |  |  |  |  |  |  |  |  |  |  |  |  |  |  |  |  |  |
| Nausea |  |  | x | x | x | x | x |  | x |  |  | x | x |  | x |  | x |  |
| Parental concern† |  |  |  |  |  |  |  |  |  |  |  |  |  |  |  |  |  |  |
| Pregnancy |  |  | x | x |  | x |  |  |  |  |  |  |  |  | x |  |  |  |
| Prior operation |  |  | x |  |  | x |  |  |  |  |  |  |  |  |  |  |  |  |
| Recent travel |  |  |  |  |  |  |  |  |  |  |  |  |  | x |  |  |  |  |
| Rectal bleeding |  |  |  | x |  | x |  |  | x |  |  |  |  |  |  |  |  |  |
| Respiratory tract infection |  |  |  | x |  | x |  |  |  |  |  |  |  |  |  |  |  |  |
| Sexual risk behavior |  |  |  |  |  | x |  |  |  |  |  |  |  |  |  |  |  |  |
| Similar pain last 6 months |  |  |  |  |  | x |  |  |  |  |  |  |  |  |  |  |  |  |
| Skin symptoms |  |  |  |  |  | x |  |  | x |  |  |  |  |  |  |  |  |  |
| Transportation pain |  |  |  |  |  | x |  |  |  |  |  |  |  |  |  |  |  |  |
| UTI, differential diagnosis |  |  |  |  |  | x |  |  |  |  |  |  |  |  |  |  |  |  |
| Vaginal bleeding |  |  |  | x |  | x |  |  |  |  |  |  |  |  |  |  |  |  |
| Voiding urgency |  |  |  |  |  | x |  |  |  |  |  |  |  |  |  |  |  |  |
| Vomiting |  |  | x | x | x | x | x |  | x |  |  | x | x |  | x |  | x | x |
| Whether pain is acute or chronic |  |  | x | x |  | x |  |  | x |  |  |  |  | x |  |  |  |  |
| **Signs** |  |  |  |  |  |  |  |  |  |  |  |  |  |  |  |  |  |  |
| Amylase† |  |  |  |  |  |  |  |  |  |  |  |  |  |  |  |  |  |  |
| Bilirubin |  |  |  | x |  |  | x | x |  |  |  |  |  |  | x |  |  |  |
| Bowel sounds absence |  |  | x | x |  | x |  |  |  |  |  | x |  |  |  |  |  |  |
| Clinical prediction rule |  |  |  | x | x |  |  |  |  |  |  | x | x |  |  |  |  |  |
| CRP |  |  | x | x |  |  | x | x | x |  |  |  |  |  | x |  |  |  |
| Differential count |  |  | x | x |  |  |  | x |  |  |  | x |  |  | x |  |  |  |
| Distension |  |  |  |  |  | x |  |  | x |  |  |  |  |  |  |  | x |  |
| Drowsiness |  |  |  |  |  | x |  |  |  |  |  |  |  |  |  |  |  |  |
| Fecal mass |  |  |  |  |  | x |  |  | x |  |  |  |  | x | x |  | x |  |
| Gait |  |  |  |  |  |  |  |  |  |  |  | x |  |  |  |  |  |  |
| General appearance |  |  |  |  |  | x |  |  |  |  |  |  |  |  |  |  |  |  |
| Guarding |  |  | x | x |  |  |  |  | x |  |  | x |  | x | x |  |  |  |
| Hemoglobin |  |  |  |  |  |  |  |  | x |  |  |  |  |  |  |  |  |  |
| Ill impression |  |  |  |  |  | x |  |  | x |  |  |  |  |  | x |  |  |  |
| Inguinal hernia |  |  |  | x |  | x |  |  | x |  |  |  |  |  |  |  | x |  |
| Inspection |  |  |  |  |  | x |  |  |  |  |  |  |  |  |  |  |  |  |
| Jaundice |  |  |  |  |  |  | x |  |  |  |  |  |  |  |  |  |  |  |
| Lipase† |  |  |  |  |  |  |  |  |  |  |  |  |  |  |  |  |  |  |
| Liver function tests† |  |  |  |  |  |  |  |  |  |  |  |  |  |  |  |  |  |  |
| Location, examination |  |  | x | x |  | x |  |  | x |  |  | x | x | x | x |  | x |  |
| Palpation |  |  | x | x |  | x |  |  | x |  |  | x |  | x | x |  |  |  |
| Percussion |  |  | x |  | x | x |  |  |  |  |  | x |  |  | x | x |  |  |
| Peritoneal irritation |  |  | x |  |  | x |  |  |  |  |  |  |  |  |  |  |  |  |
| Psoas sign |  |  | x | x |  |  |  |  |  |  |  |  |  |  | x |  |  |  |
| Purpura |  |  |  |  |  | x |  |  | x |  |  |  |  |  |  |  |  |  |
| Rebound tenderness |  |  |  | x |  |  |  |  |  |  |  | x | x |  |  | x |  |  |
| Rectal abnormalities |  |  |  | x |  | x |  |  | x |  |  |  |  |  |  |  |  |  |
| Rectal examination |  |  |  | x |  | x |  |  | x |  |  |  |  |  |  |  |  |  |
| Rovsing's sign |  |  | x | x |  |  |  |  |  |  |  |  |  |  | x |  |  |  |
| Scars |  |  | x |  |  |  |  |  |  |  |  |  |  |  |  |  |  |  |
| Scrotal tenderness/swelling |  |  |  | x |  | x |  |  | x |  |  |  |  |  |  |  |  |  |
| Swelling |  |  |  |  |  | x |  |  | x |  |  |  |  |  |  |  |  |  |
| Temperature |  |  | x | x | x | x |  |  |  |  |  | x | x | x | x |  | x |  |
| Tenderness |  |  | x | x | x |  | x |  | x |  |  | x | x | x | x | x | x |  |
| Urinalysis§ |  |  | x | x |  | x |  |  | x |  |  |  |  |  |  |  | x |  |
| WBC |  |  |  | x |  |  |  | x | x |  |  | x |  | x | x |  |  |  |
| ICPC diagnostic code |  |  |  |  |  |  |  |  |  |  |  |  |  |  |  |  |  |  |
| Differential diagnosis | x | x |  |  |  |  |  |  | x |  | x |  |  | x |  |  |  |  |
| **Advice for next physician** |  |  |  |  |  |  |  |  |  |  |  |  |  |  |  |  |  |  |
| Alarm symptoms |  |  |  |  |  | x |  |  |  |  |  |  |  |  |  |  |  |  |
| Expected course |  |  |  |  |  |  |  |  |  | x | x |  |  |  |  |  |  |  |
| Find help when needed |  | x |  |  |  |  |  |  |  | x |  |  |  |  |  |  |  |  |
| Give patient flyer |  | x |  |  |  |  |  |  |  | x | x |  |  |  |  |  |  | x |
| How follow-up will take place |  | x |  |  |  |  |  |  |  | x | x |  |  |  |  |  |  | x |
| How to find help |  | x |  |  |  |  |  |  |  | x | x |  |  |  |  |  |  | x |
| Options for follow-up discussed |  | x |  |  |  |  |  |  |  |  |  |  |  |  |  |  |  |  |
| Own feelings | x |  |  |  |  |  |  |  |  |  |  |  |  |  |  |  |  |  |
| Peer consultation | x |  |  |  |  |  |  |  |  |  |  |  |  |  |  |  |  |  |
| Reaction of patient | x |  |  |  |  |  |  |  |  |  |  |  |  |  |  |  |  |  |
| Safety net advice given | x | x |  |  |  |  |  |  |  | x | x |  |  |  |  |  |  | x |
| Uncertainty of diagnosis | x | x |  |  |  |  |  |  |  | x | x |  |  |  |  |  |  | x |

Abbreviations: CPR, clinical prediction rule; CRP, C-reactive protein; GP, general practitioner; ICPC, international classification of primary care; NSAID, non-steroidal anti-inflammatory drugs; POCT, point-of-care testing; UTI, Urinary tract infection; WBC, white blood count.

†, suggested by participants; ^§^, mentioned in Dutch guideline, omitted in Round 1.
