# Supplementary material for: What is important to the GP in recognizing acute appendicitis in children: a delphi study
Source: BMC Prim Care. 2023 Oct 23;24:217. doi: 10.1186/s12875-023-02167-6 (PMC10591392; doi:10.1186/s12875-023-02167-6)
Supplement: Supplementary file 5 — Supplementary Material 5 [file 12875_2023_2167_MOESM5_ESM.docx]

**Appendix 5.** Percentages of participants considering requirements as important or not in each Delphi round.

| **Feature (sign/symptom)** | **Requirement** | **Is the requirement considered important?** | | | | | |
| --- | --- | --- | --- | --- | --- | --- | --- |
|  |  | **Round 1** | | **Round 2** | | **Round 3** | |
|  |  | **No (%)** | **Yes (%)** | **No (%)** | **Yes (%)** | **No (%)** | **Yes (%)** |
| **Symptoms** |  |  |  |  |  |  |  |
| Abdominal pain | Ask about symptom | 0 | 100 | 0 | 100 |  |  |
|  | Record presence | 0 | 100 | 0 | 100 |  |  |
|  | Record absence | 9.1 | 90.9 | 4.5 | 95.5 |  |  |
| Analgesic use | Ask about symptom | 9.1 | 90.9 | 18.2 | 81.8 |  |  |
| *Paracetamol* | Record presence | 22.7 | 77.3 | 13.6 | 86.4 |  |  |
| *Paracetamol* | Record absence | 50 | 50 | 54.5 | 45.5 |  |  |
| *NSAID* | Record presence | 22.7 | 77.3 | 9.5 | 90.5 |  |  |
| *NSAID* | Record absence | 68.2 | 31.8 | 77.3 | 22.7 |  |  |
| *Opioid* | Record presence | 27.3 | 72.7 | 9.1 | 90.9 |  |  |
| *Opioid* | Record absence | 90.9 | 9.1 |  |  |  |  |
| Anorexia | Ask about symptom | 40.9 | 59.1 | 50 | 50 |  |  |
|  | Record presence | 40.9 | 59.1 | 38.1 | 61.9 |  |  |
|  | Record absence | 54.5 | 45.5 | 59.1 | 40.9 |  |  |
| Blunt abdominal trauma | Ask about symptom | 77.3 | 22.7 |  |  |  |  |
|  | Record presence | 31.8 | 68.2 | 9.1 | 90.9 | 0 | 100 |
|  | Record absence | 100 | 0 |  |  |  |  |
| Chronic disease | Ask about symptom | 31.8 | 68.2 | 31.8 | 68.2 |  |  |
|  | Record presence | 27.3 | 72.7 | 18.2 | 81.8 |  |  |
|  | Record absence | 86.4 | 13.6 |  |  |  |  |
| Constipation | Ask about symptom | 13.6 | 86.4 | 9.1 | 90.9 |  |  |
|  | Record presence | 13.6 | 86.4 | 0 | 100 |  |  |
|  | Record absence | 50 | 50 | 54.5 | 45.5 |  |  |
| Coughing pain | Ask about symptom | 63.6 | 36.4 | 68.2 | 31.8 |  |  |
|  | Record presence | 50 | 50 | 42.9 | 57.1 |  |  |
|  | Record absence | 77.3 | 22.7 |  |  |  |  |
| Diarrhea | Ask about symptom | 18.2 | 81.8 | 9.1 | 90.9 |  |  |
|  | Record presence | 22.7 | 77.3 | 4.5 | 95.5 |  |  |
|  | Record absence | 59.1 | 40.9 | 63.6 | 36.4 |  |  |
| Duration of pain | Ask about symptom | 0 | 100 | 4.5 | 95.5 |  |  |
|  | Record finding | 0 | 100 | 0 | 100 |  |  |
| Dysmenorrhea | Ask about symptom | 31.8 | 68.2 | 13.6 | 86.4 | 50 | 50 |
| Dysuria | Ask about symptom | 9.1 | 90.9 | 23.8 | 76.2 |  |  |
|  | Record presence | 18.2 | 81.8 | 9.1 | 90.9 |  |  |
|  | Record absence | 68.2 | 31.8 | 54.5 | 45.5 |  |  |
| Fecal incontinence ^a^ | Ask about symptom | 90.9 | 9.1 |  |  |  |  |
|  | Record presence | 77.3 | 22.7 |  |  |  |  |
|  | Record absence | 95.5 | 4.5 |  |  |  |  |
| Fever | Ask about symptom | 9.1 | 90.9 | 4.5 | 95.5 |  |  |
|  | Record presence | 22.7 | 77.3 | 0 | 100 |  |  |
|  | Record absence | 27.3 | 72.7 | 4.5 | 95.5 |  |  |
| Fluid intake | Ask about symptom | 40.9 | 59.1 | 31.8 | 68.2 |  |  |
|  | Record presence | 27.3 | 72.7 | 36.4 | 63.6 |  |  |
|  | Record absence | 72.7 | 27.3 |  |  |  |  |
| Frequency of bowel movements | Ask about symptom | 36.4 | 63.6 | 40.9 | 59.1 |  |  |
| *Decreased* | Record presence | 54.5 | 45.5 | 40.9 | 59.1 |  |  |
| *Increased* | Record presence | 50 | 50 | 36.4 | 63.6 |  |  |
| *Decreased* | Record absence | 63.6 | 36.4 | 77.3 | 22.7 |  |  |
| *Increased* | Record absence | 81.8 | 18.2 |  |  |  |  |
| Frequent micturition | Ask about symptom | 36.4 | 63.6 | 31.8 | 68.2 |  |  |
|  | Record presence | 36.4 | 63.6 | 18.2 | 81.8 | 27.2 | 72.7 |
|  | Record absence | 86.4 | 13.6 |  |  |  |  |
| Hematuria | Ask about symptom | 63.6 | 36.4 | 61.9 | 38.1 |  |  |
|  | Record presence | 31.8 | 68.2 | 13.6 | 86.4 | 9.1 | 90.9 |
|  | Record absence | 95.5 | 4.5 |  |  |  |  |
| IBS | Ask about symptom | 54.5 | 45.5 | 68.2 | 31.8 |  |  |
|  | Record presence | 45.5 | 54.5 | 36.4 | 63.6 |  |  |
|  | Record absence | 86.4 | 13.6 |  |  |  |  |
| Illness in family | Ask about symptom |  |  | 40.9 | 59.1 | 40.9 | 59.1 |
|  | Record finding |  |  | 57.1 | 42.9 | 63.6 | 36.4 |
| Intensity of pain | Ask about symptom | 13.6 | 86.4 | 13.6 | 86.4 |  |  |
|  | Record finding | 27.3 | 72.7 | 31.8 | 68.2 |  |  |
| Joint pain | Ask about symptom | 81.8 | 18.2 |  |  |  |  |
|  | Record presence | 40.9 | 59.1 | 50 | 50 |  |  |
|  | Record absence | 100 | 0 |  |  |  |  |
| Last use of analgesics | Ask about symptom | 22.7 | 77.3 | 40.9 | 59.1 |  |  |
|  | Record finding | 59.1 | 40.9 | 50 | 50 |  |  |
| Location of pain | Ask about symptom | 4.5 | 95.5 | 0 | 100 |  |  |
|  | Record finding | 0 | 100 | 0 | 100 |  |  |
| Low back pain | Ask about symptom | 54.5 | 45.5 | 77.3 | 22.7 |  |  |
|  | Record presence | 95.5 | 4.5 |  |  |  |  |
|  | Record absence | 22.7 | 77.3 | 40.9 | 59.1 |  |  |
| Menstrual cycle abnormal | Ask about symptom | 22.7 | 77.3 | 4.5 | 95.5 |  |  |
|  | Record presence | 22.7 | 77.3 | 13.6 | 86.4 |  |  |
|  | Record absence | 81.8 | 18.2 |  |  |  |  |
| Migration of pain | Ask about symptom | 22.7 | 77.3 | 22.7 | 77.3 |  |  |
|  | Record presence | 31.8 | 68.2 | 40.9 | 59.1 |  |  |
|  | Record absence | 59.1 | 40.9 | 77.3 | 22.7 |  |  |
| Nature of pain | Ask about symptom |  |  | 31.8 | 68.2 | 18.2 | 81.8 |
|  | Record finding |  |  | 36.4 | 63.6 | 13.6 | 86.4 |
| Nausea | Ask about symptom | 31.8 | 68.2 | 28.6 | 71.4 | 22.7 | 77.3 |
|  | Record presence | 27.3 | 72.7 | 13.6 | 86.4 |  |  |
|  | Record absence | 45.5 | 54.5 | 45.5 | 54.5 |  |  |
| Parental concern | Record finding |  |  | 18.2 | 81.8 | 36.4 | 63.6 |
| Pregnancy | Ask about symptom | 4.5 | 95.5 | 9.1 | 90.9 |  |  |
|  | Record presence | 4.5 | 95.5 | 0 | 100 |  |  |
|  | Record absence | 31.8 | 68.2 | 22.7 | 77.3 | 31.8 | 68.2 |
| Prior operation | Ask about symptom | 18.2 | 81.8 | 13.6 | 86.4 |  |  |
|  | Record presence | 18.2 | 81.8 | 9.1 | 90.9 |  |  |
|  | Record absence | 90.9 | 9.1 |  |  |  |  |
| Recent travel | Ask about symptom | 72.7 | 27.3 |  |  |  |  |
|  | Record presence | 36.4 | 63.6 | 36.4 | 63.6 |  |  |
|  | Record absence | 86.4 | 13.6 |  |  |  |  |
| Rectal bleeding | Ask about symptom | 40.9 | 59.1 | 42.9 | 57.1 |  |  |
|  | Record presence | 9.1 | 90.9 | 9.1 | 90.9 |  |  |
|  | Record absence | 59.1 | 40.9 | 66.7 | 33.3 |  |  |
| RTI | Ask about symptom | 59.1 | 40.9 | 63.6 | 36.4 |  |  |
|  | Record presence | 50 | 50 | 40.9 | 59.1 |  |  |
|  | Record absence | 90.9 | 9.1 |  |  |  |  |
| Sexual risk behavior | Ask about symptom | 31.8 | 68.2 | 18.2 | 81.8 | 27.3 | 72.7 |
|  | Record presence | 27.3 | 72.7 | 22.7 | 77.3 |  |  |
|  | Record absence | 63.6 | 36.4 | 63.6 | 36.4 |  |  |
| Similar pain last 6 months | Ask about symptom | 22.7 | 77.3 | 36.4 | 63.6 |  |  |
|  | Record presence | 40.9 | 59.1 | 40.9 | 59.1 |  |  |
| Skin symptoms | Ask about symptom | 100 | 0 |  |  |  |  |
|  | Record presence | 68.2 | 31.8 | 50 | 50 |  |  |
|  | Record absence | 100 | 0 |  |  |  |  |
| Transportation pain | Ask about symptom | 4.5 | 95.5 | 9.1 | 90.9 |  |  |
|  | Record presence | 9.1 | 90.9 | 0 | 100 |  |  |
|  | Record absence | 27.3 | 72.7 | 27.3 | 72.7 |  |  |
| UTI dd | Ask about symptom | 13.6 | 86.4 | 22.7 | 77.3 |  |  |
| Vaginal bleeding | Ask about symptom | 36.4 | 63.6 | 31.8 | 68.2 |  |  |
|  | Record presence | 36.4 | 63.6 | 18.2 | 81.8 | 9.1 | 90.9 |
|  | Record absence | 86.4 | 13.6 |  |  |  |  |
| Voiding urgency | Ask about symptom | 68.2 | 31.8 | 90.9 | 9.1 |  |  |
|  | Record presence | 54.5 | 45.5 | 66.7 | 33.3 |  |  |
|  | Record absence | 95.5 | 4.5 |  |  |  |  |
| Vomiting | Ask about symptom | 31.8 | 68.2 | 9.1 | 90.9 | 18.2 | 81.8 |
|  | Record presence | 18.2 | 81.8 | 0 | 100 |  |  |
|  | Record absence | 36.4 | 63.6 | 31.8 | 68.2 |  |  |
| Whether pain is acute or chronic | Ask about symptom | 0 | 100 | 22.7 | 77.3 |  |  |
|  | Record finding | 0 | 100 | 13.6 | 86.4 |  |  |
| **Signs** |  |  |  |  |  |  |  |
| Amylase | Examine sign |  |  | 95.5 | 4.5 |  |  |
| Bilirubin | Examine sign | 95.5 | 4.5 |  |  |  |  |
| Bowel sounds auscultation | Examine sign | 18.2 | 81.8 | 18.2 | 81.8 |  |  |
| *Absence* | Record finding | 4.5 | 95.5 | 0 | 100 |  |  |
| *Decreased* | Record finding | 22.7 | 77.3 | 31.8 | 68.2 |  |  |
| *Increased* | Record finding | 31.8 | 68.2 | 31.6 | 68.4 |  |  |
| *Normal* | Record finding | 27.3 | 72.7 | 38.1 | 61.9 |  |  |
| Clinical prediction rule | Examine sign | 63.6 | 36.4 | 66.7 | 33.3 |  |  |
|  | Record presence | 54.5 | 45.5 | 31.8 | 68.2 |  |  |
|  | Record absence | 68.2 | 31.8 | 68.2 | 31.8 |  |  |
| CRP | Examine sign | 13.6 | 86.4 | 59.1 | 40.9 |  |  |
| *Laboratory* | Examine sign | 63.6 | 36.4 | 86.4 | 13.6 |  |  |
| *POCT* | Examine sign | 18.2 | 81.8 | 22.7 | 77.3 |  |  |
| *Result* | Record finding | 4.5 | 95.5 | 9.5 | 90.5 |  |  |
| Differential count | Examine sign | 90.9 | 9.1 |  |  |  |  |
| Distension | Record presence | 4.5 | 95.5 | 18.2 | 81.8 |  |  |
|  | Record absence | 81.8 | 18.2 |  |  |  |  |
| Drowsiness | Record presence | 0 | 100 | 0 | 100 |  |  |
|  | Record absence | 40.9 | 59.1 | 47.6 | 52.4 |  |  |
| Fecal mass | Record presence | 9.1 | 90.9 | 4.5 | 95.5 |  |  |
|  | Record absence | 77.3 | 22.7 |  |  |  |  |
| Gait | Examine sign | 18.2 | 81.8 | 18.2 | 81.8 |  |  |
| *Abnormal* | Record finding | 72.7 | 27.3 |  |  |  |  |
| *Difficulty walking* | Record finding | 18.2 | 81.8 | 27.3 | 72.7 |  |  |
| *Not able to walk* | Record finding | 27.3 | 72.7 | 13.6 | 86.4 |  |  |
| General appearance | Examine sign | 0 | 100 | 0 | 100 |  |  |
| Guarding | Record presence | 0 | 100 | 0 | 100 |  |  |
|  | Record absence | 13.6 | 86.4 | 22.7 | 77.3 |  |  |
|  | Record finding | 36.4 | 63.6 | 50 | 50 |  |  |
| Hemoglobin | Examine sign | 90.9 | 9.1 |  |  |  |  |
| Heel-drop sign | Record absence | 77.3 | 22.7 |  |  |  |  |
|  | Examine sign |  |  | 68.2 | 31.8 | 81.8 | 18.2 |
|  | Record presence | 54.5 | 45.5 | 50 | 50 |  |  |
| Ill impression | Record presence | 0 | 100 | 4.5 | 95.5 |  |  |
|  | Record absence | 13.6 | 86.4 | 13.6 | 86.4 |  |  |
| Inguinal hernia | Examine sign | 50 | 50 | 59.1 | 40.9 |  |  |
|  | Record presence | 18.2 | 81.8 | 0 | 100 |  |  |
|  | Record absence | 68.2 | 31.8 |  |  | 81.8 | 18.2 |
| Inspection | Examine sign | 22.7 | 77.3 | 22.7 | 77.3 |  |  |
| Jaundice | Examine sign | 68.2 | 31.8 | 72.7 | 27.3 |  |  |
|  | Record presence | 22.7 | 77.3 | 4.5 | 95.5 |  |  |
|  | Record absence | 90.9 | 9.1 |  |  |  |  |
| Lipase | Examine sign |  |  | 95.5 | 4.5 |  |  |
| Liver function tests | Examine sign |  |  | 90.9 | 9.1 |  |  |
| Location of pain, pointing | Examine sign | 4.5 | 95.5 | 9.1 | 90.9 |  |  |
|  | Record finding | 4.5 | 95.5 | 45.5 | 54.5 |  |  |
| Location of pain, examination | Record finding | 0 | 100 | 0 | 100 |  |  |
| Palpation | Examine sign | 0 | 100 | 0 | 100 |  |  |
| Percussion | Examine sign | 13.6 | 86.4 | 22.7 | 77.3 |  |  |
| Percussion Tenderness | Record presence | 0 | 100 | 18.2 | 81.8 |  |  |
| Percussion Tenderness | Record absence | 31.8 | 68.2 | 42.9 | 57.1 |  |  |
| Peritoneal irritation | Record presence | 0 | 100 | 0 | 100 |  |  |
|  | Record absence | 27.3 | 72.7 | 27.3 | 72.7 |  |  |
| Psoas sign | Examine sign | 36.4 | 63.6 | 31.8 | 68.2 |  |  |
|  | Record presence | 18.2 | 81.8 | 13.6 | 86.4 |  |  |
|  | Record absence | 68.2 | 31.8 | 81.8 | 18.2 |  |  |
| Purpura | Examine sign | 50 | 50 | 54.5 | 45.5 |  |  |
|  | Record presence | 22.7 | 77.3 | 4.5 | 95.5 |  |  |
|  | Record absence | 90.9 | 9.1 |  |  |  |  |
| Rebound tenderness | Record presence | 0 | 100 | 4.5 | 95.5 |  |  |
|  | Record absence | 18.2 | 81.8 | 27.3 | 72.7 |  |  |
| Rectal abnormalities* | Record presence | 50 | 50 |  |  |  |  |
|  | Record absence | 86.4 | 13.6 |  |  |  |  |
| Rectal examination | Examine sign | 90.9 | 9.1 |  |  |  |  |
|  | Record presence | 68.2 | 31.8 |  |  |  |  |
| Rectal fecal mass* | Record absence | 81.8 | 18.2 |  |  |  |  |
| Rovsing’s sign | Examine sign | 36.4 | 63.6 | 40.9 | 59.1 |  |  |
|  | Record presence | 36.4 | 63.6 | 31.8 | 68.2 |  |  |
|  | Record absence | 72.7 | 27.3 |  |  |  |  |
| Scars | Record presence | 9.1 | 90.9 | 22.7 | 77.3 |  |  |
|  | Record absence | 95.5 | 4.5 |  |  |  |  |
| Scrotal tenderness/swelling | Examine sign | 54.5 | 45.5 | 45.5 | 54.5 |  |  |
|  | Record presence | 18.2 | 81.8 | 4.5 | 95.5 |  |  |
|  | Record absence | 77.3 | 22.7 |  |  |  |  |
| Summarize as “abdomen completely soft without tenderness” | Record finding |  |  | 27.3 | 72.7 | 13.6 | 86.4 |
| Summarize: no abnormalities | Record finding | 68.2 | 31.8 | 59.1 | 40.9 |  |  |
| Swelling | Record presence | 4.5 | 95.5 | 0 | 100 |  |  |
|  | Record absence | 59.1 | 40.9 | 86.4 | 13.6 |  |  |
| Temperature | Examine sign | 0 | 100 | 4.5 | 95.5 |  |  |
|  | Record finding | 0 | 100 | 0 | 100 |  |  |
| Tenderness | Record presence | 4.5 | 95.5 | 4.5 | 95.5 |  |  |
|  | Record absence | 13.6 | 86.4 | 18.2 | 81.8 |  |  |
| Urinalysis | Examine sign |  |  | 27.3 | 72.7 | 4.5 | 95.5 |
| WBC | Examine sign | 72.7 | 27.3 |  |  |  |  |
| Differential diagnosis | Record finding | 18.2 | 81.8 | 22.7 | 77.3 |  |  |
| **Diagnosis** |  |  |  |  |  |  |  |
| ICPC diagnostic code | Record finding | 86.4 | 13.6 |  |  |  |  |
| ICPC symptom code | Record finding | 18.2 | 81.8 | 13.6 | 86.4 |  |  |
| **Actions** |  |  |  |  |  |  |  |
| Advice for next physician | Record advice | 13.6 | 86.4 | 22.7 | 77.3 |  |  |
| Alarm symptoms | Explain | 0 | 100 | 9.1 | 90.9 |  |  |
|  | Record advice | 31.8 | 68.2 | 18.2 | 81.8 | 27.3 | 72.7 |
| Expected course | Action | 0 | 100 | 22.7 | 77.3 |  |  |
|  | Record advice | 40.9 | 59.1 | 36.4 | 63.6 |  |  |
| Find help when needed | Action | 0 | 100 | 9.1 | 90.9 |  |  |
|  | Record advice | 27.3 | 72.7 | 22.7 | 77.3 |  |  |
| Give patient flyer | Act | 90.9 | 9.1 |  |  |  |  |
|  | Record action | 72.7 | 27.3 |  |  |  |  |
| How follow-up will take place | Record action | 13.6 | 86.4 | 18.2 | 81.8 |  |  |
| How to find help | Explain | 9.1 | 90.9 | 18.2 | 81.8 |  |  |
| How to find help | Record advice | 59.1 | 40.9 | 40.9 | 59.1 |  |  |
| Options for follow-up | Record advice | 13.6 | 86.4 | 19.0 | 81.0 |  |  |
| Own feelings | Record finding | 31.8 | 68.2 | 9.1 | 90.9 | 50 | 50 |
| Peer consultation | Record presence | 9.1 | 90.9 | 13.6 | 86.4 |  |  |
|  | Record absence | 90.9 | 9.1 |  |  |  |  |
| Reaction of patient | Record finding | 31.8 | 68.2 | 19 | 81 | 72.7 | 27.3 |
| Safety net advice given | Record advice | 36.4 | 63.6 | 22.7 | 77.3 | 40.9 | 59.1 |
| Uncertainty of diagnosis | Explain | 0 | 100 | 9.1 | 90.9 |  |  |
|  | Record advice | 40.9 | 59.1 | 27.3 | 72.7 | 50 | 50 |

Abbreviations: CRP, C-reactive protein; dd, differential diagnosis; IBS, irritable bowel syndrome; RTI, respiratory tract infection; UTI, urinary tract infection.

Fields are marked orange when the requirement was on the concept consensus list. Percentages are marked green when the requirement reached consensus and was added to the final consensus list. For requirements not on the consensus list: the percentage for “not important” represents participants who rated the requirement not important at all, not important or neutral; and the percentage for “important” represents those who rated the requirement as important or very important. Note: ^*^ Performing rectal examination was rated as not important, so the three related requirements (rectal abnormalities presence/absence and fecal mass presence) were removed.
