# Supplementary material for: What is important to the GP in recognizing acute appendicitis in children: a delphi study
Source: BMC Prim Care. 2023 Oct 23;24:217. doi: 10.1186/s12875-023-02167-6 (PMC10591392; doi:10.1186/s12875-023-02167-6)
Supplement: Supplementary file 6 — Supplementary Material 6 [file 12875_2023_2167_MOESM6_ESM.docx]

**Appendix 6.** Qualitative feedback by participants.

| Subject | Comment | No. |
| --- | --- | --- |
| Symptoms | - The GP will ask an open-ended question instead of asking about all symptoms. | 1 |
|  | - Symptoms asked by the GP depend on differential diagnosis. | 1 |
| Parental concern | - GPs will record parental concern only when concern is high. | 1 |
|  | - GPs will record patient reaction only when patient does not agree. | 3 |
| Signs | - Signs investigated depend on symptoms. E.g., eliciting pain may cause stress but add little information. | 1 |
|  | - Examination of abdomen should not be summarized as: “no abnormalities routinely.” However, this is common practice. | 1 |
|  | - Abnormalities are not especially searched for, but they are important when found. | 2 |
|  | - Some sings always warrant referral and cannot occur in a child that needs reassessment, making some questions incompatible with the case description. | 2 |
| Test | - Urinalysis should be done only if relevant to differential diagnosis. | 1 |
|  | - CRP-testing should be carried out routinely when doing a planned reassessment. | 1 |
| Diagnosis | - Use of diagnostic codes depends on degree of suspicion and do not always reflect confirmed diagnoses. | 1 |
| Peer consultation | - Peer consultation should be avoided by the GP. When suspicion is high enough for consulting a colleague, referral is always necessary. | 1 |
| General | - Medical records are not important for reassessment. The second doctor cannot rely on records but only on own observation. | 2 |
|  | - It appears as if a legal document is made in case something goes wrong. | 1 |
| Questionnaire | - Answers cannot always be expressed as important or not important. | 1 |
|  | - The initial list much too long. | 3 |
|  | - The questionnaire is confusing, and therefore not reproducible on participant-level. | 1 |

Comments by participants are presented by subject with the number of times the same comment was made.
